# Genome-Wide RNAi Screening Identifies Genes Inhibiting the Migration of Glioblastoma Cells

**DOI:** 10.1371/journal.pone.0061915

**Published:** 2013-04-12

**Authors:** Jian Yang, Jing Fan, Ying Li, Fuhai Li, Peikai Chen, Yubo Fan, Xiaofeng Xia, Stephen T. Wong

**Affiliations:** Department of Systems Medicine and Bioengineering, The Methodist Hospital Research Institute, Weill Cornell Medical College, Houston, Texas, United States of America; Wake Forest University, School of Medicine, United States of America

## Abstract

Glioblastoma Multiforme (GBM) cells are highly invasive, infiltrating into the surrounding normal brain tissue, making it impossible to completely eradicate GBM tumors by surgery or radiation. Increasing evidence also shows that these migratory cells are highly resistant to cytotoxic reagents, but decreasing their migratory capability can re-sensitize them to chemotherapy. These evidences suggest that the migratory cell population may serve as a better therapeutic target for more effective treatment of GBM. In order to understand the regulatory mechanism underlying the motile phenotype, we carried out a genome-wide RNAi screen for genes inhibiting the migration of GBM cells. The screening identified a total of twenty-five primary hits; seven of them were confirmed by secondary screening. Further study showed that three of the genes, FLNA, KHSRP and HCFC1, also functioned *in vivo*, and knocking them down caused multifocal tumor in a mouse model. Interestingly, two genes, KHSRP and HCFC1, were also found to be correlated with the clinical outcome of GBM patients. These two genes have not been previously associated with cell migration.

## Introduction

Glioblastoma multiforme (GBM) is the most common form of primary brain tumor in adults [1], [2]. Despite years of effort, the life expectancy for GBM patients has not improved significantly, with an average of about only 15 months [3]. In the US, approximately 15,000 patients die from GBM every year. The poor prognosis partly originates from GMB's invasive phenotype, which gives the tumor cells the ability to infiltrate into adjacent normal brain tissue. In pathology, a penumbra of invasive single cells can often be detected several centimeters from the core lesion. This has made it extremely difficult to completely eradicate a tumor by traditional treatment modalities such as surgical resection or radiation [4], [5]. As a result tumors frequently recur and none of the current treatment options are ultimately effective [6]. Also notably, although the invasiveness does not necessarily correlate with the grade of malignancy for gliomas [7], it has been shown that invasive GBM cells may have heightened resistance to the induction of apoptosis [8]. Therefore, chemotherapy is often ineffective on these cells, further contributing to GBM's poor prognosis. Interestingly, decreasing the migratory capabilities of tumor cells can restore a certain level of sensitivity to cytotoxic reagents and increase the susceptibility to chemotherapeutic treatments [9], [10]. These results suggest that the invasive cell population may represent a more effective treatment target for GBM.

Tumor invasion is the result of a complex interaction of cancer cells with the surrounding structures. It begins with individual cell migration, a process that is driven by the cytoskeleton rearrangement and the focal adhesion assembly [11], [12]. Cell migration is involved in many normal physiological processes, such as embryonic development, wound healing, and inflammatory response [13], [14], [15]. It is believed to be a rigidly controlled process that is under the regulation of complex mechanisms mediated by numerous genes. Cells of origin of GBM, be it astrocytes or stem/progenitor cells, are intrinsically migratory. However, the migratory capability of tumor cells varies among patients. It is possible that the enhanced motile phenotype of GBM cells is caused by the lost of one or more regulatory controls, as a direct or indirect result of the numerous somatic mutations that are frequently observed in GBM [16]. Although much has been learned about the phenotypic profile of cell migration in GBM, little is known about its causing mechanism. Characterizing the molecular mechanisms may not only provide better diagnostic and prognostic biomarkers, but also discover novel molecular therapeutic targets.

To shed light on the mechanism that drives GBM tumor invasion and to identify novel molecular targets that can possibly be used for disease management, we sought to systematically characterize the genes inhibiting the migration of GBM cells. To this end we adopted a pooled genome-wide RNA interference (RNAi) screening approach [17]. RNAi knocks down the RNA target in a sequence-specific manner and greatly facilitates the study of individual genes [18], [19], [20]. Paired with genomic sequence data, high-throughput RNAi screening is now possible, allowing systematic functional analysis on a genome-wide scale [21], [22], [23]. Using this unbiased approach, we successfully identified a number of genes that were later confirmed to regulate GBM cell migration both *in vitro* and *in vivo*. Further investigation showed that two of these genes are also associated with the clinical outcome of GBM patients.

## Methods

### Ethics statement

Brain tumor surgical specimens were obtained following the protocol approved by Methodist Hospital Institutional Review Board (IRB0907-0187). Tissue samples were obtained by The Methodist Hospital Tissue Bank from patients with signed consent forms, the samples were provided to us by the tissue bank without any of the patient's identity information. All animal experiments were performed following the protocol approved by The Methodist Hospital Institutional Animal Care and Use Committee (AUP-0811-0037). All surgery was performed under Ketamine/Xylazine cocktail anesthesia, and all efforts were made to minimize suffering.

### Cell lines and primary cells

All the cell lines were purchased from the American Type Culture Collection (ATCC) and grown in DMEM with 10% fetal bovine serum (FBS) and penicillin/streptomycin at 37°C in a humidified incubator with 5% CO_2_. They were used within 10 passages, for less than 6 months after receipt. Cell lines were characterized by ATCC by morphology check, growth curve analysis, and short tandem repeat DNA profiling. After receipt, cells were confirmed to be free from mycoplasma contamination using a mycoplasma detection kit (Roche Applied Science).

Brain tumor surgical specimen were obtained following the protocol approved by our Institutional Review Board. Briefly, fresh tumor samples obtained within 2 hours of surgical resection were rinsed with PBS, mechanically minced with scissors, and digested for 30 minutes at 37°C with trypsin. Cells were extensively triturated and filtered through a 40 µm filter to collect single cells. They were then cultured in suspension at 10^5^ cells/ml in serum free medium containing bFGF, EGF, and heparin. Neurospheres formed within a week and the single cells were removed using cell strainers. The cells were maintained in the neurosphere form and used for migration assay within 2 weeks. Before the assay the neurospheres were dissociated with accutase to single cells.

### Lentivirus transduction

The Decode RNAi viral screening kit was purchased from Open Biosystems. Virus was provide as high-titer pre-packaged lentiviral particles produced from a pGIPZ vector. The shRNA sequences were designed to be microRNA-adapted to enhance the efficiency and each construct was barcoded for identification. For transduction, 1.5×10^6^ U87 cells were plated in a 100 mm dish. The next day, the medium was replaced with 3 ml virus containing medium. After 6 hours incubation, the virus was removed and the cells were further cultured in fresh medium for 48 hours. Non-transduced cells were then removed by incubating the cells in puromycin containing medium for 6 days. The transduction rate was monitored by the expression of GFP. As a negative control, mock transduced cells were prepared by transducing with virus from the same lentiviral vector harboring a scrambled shRNA sequence (Open biosystems Catalog# RHS4346).

To build the overexpressing cell lines, the coding sequences of the targeting genes were cloned into a pLentif6/V5 (Life Technologies) vector and lentivirus was prepared following the manufacturer's instruction. After infection non-transduced cells were removed by antibiotic selection.

### Microarray hybridization

Cells of interest were collected and genomic DNA was extracted using a Promega kit. A 250 base pair DNA fragment containing the barcode sequence was PCR amplified using the primers supplied by the vendor (Open Biosystems). The DNA was then labeled with cyanine fluorescent dyes using Agilent's genomic DNA labeling kit. The labeled DNAs were then hybridized to microarray using the Agilent oligo aCGH hybridization kit, and the array was scanned with a Genepix scanner. The Decode RNAi barcode microarray was supplied by the vendor (Open Biosystems). It consists of 2×105 K arrays spotted with sequences unique to each shRNA. Barcode hybridizing probes have been optimized using Agilent algorithms for assessing probe quality. Each array contains 58,498 probe sequences (most duplicated on the array).

### Cell migration assays

For Boyden chamber assay, experiments were carried out using Matrigel invasion chambers with 8 µm pore size (BD Biosciences). To count the migrated cells, after incubation the non-invading cells were thoroughly removed from the upper surface of the membrane by scrubbing. The migrated cells attached to the lower surface of the membrane were fixed and stained with toluidine blue. The whole membrane was then imaged using a brightfield microscope with montage function. For microarray analysis or further culture, cells were separately collected from the upper chamber and lower membrane surface, trypsinized, and washed for further treatment. For wound healing assay, 5×10^5^ cells were seeded in 6-well plates. After 48 hours, a straight scratch was made in each well using a pipette tip. Time-lapse images were taken and the migrated cells were counted at different time points. All experiments were repeated at least three times, and results were presented as the mean with standard deviation. Student T test was used to evaluate the statistical significance.

### Cell proliferation assay

To measure the cell proliferation rate, 1×10^4^ cells were seeded in a well of 24-well plate. Every 24 hours, cell proliferation was measured using a MTS assay kit (Promega) for 6 days. As confirmation viable cell count was also carried out to measure cell proliferation. 1.5×10^3^ cells were seeded in a well of 96-well plate. Every 24 hours cells were dissociated and viable cells free of tryphan blue staining were counted until 6 days later. For both experiments results are presented as the mean of 6 independent wells with standard deviation. Student T test was used to evaluate the statistical significance.

### Cell-matrix, cell-cell adhesion assay

A Vibrant Cell Adhesion Assay kit (Life Technologies) was used to examine the cell-matrix adhesion. Cells were stained with calcein AM before they were plated into a Matrigel coated 96-well plate, after 1 hour non-adherent cells were removed by careful washing, and the adherent cells were quantified by measuring the fluorescence intensity using a plate reader. Similarly, to measure cell-cell interaction, calcein AM stained U87 cells were plated into wells that were already covered with U87 cells. After 1 hour the well was washed and fluorescence intensity was measured to determine the number of adherent cells.

### Mouse tumor model

Immunodeficient NOD/SCID mice were purchased from Charles River and experiments were carried out in accordance with the institutional guidelines for the use of laboratory animals. 200,000 transduced U87 cells were suspended in 10 µl sterile PBS for injection. Cells were implanted subcortically into the right hemisphere (2 mm lateral, 2 mm in front of bregma, and 2 mm deep) using a stereotactic fixation device. After the animals died from tumor, the brains were dissected and H/E stained for pathology examination.

## Results

### Genome-wide RNAi screening

**Figure 1:**
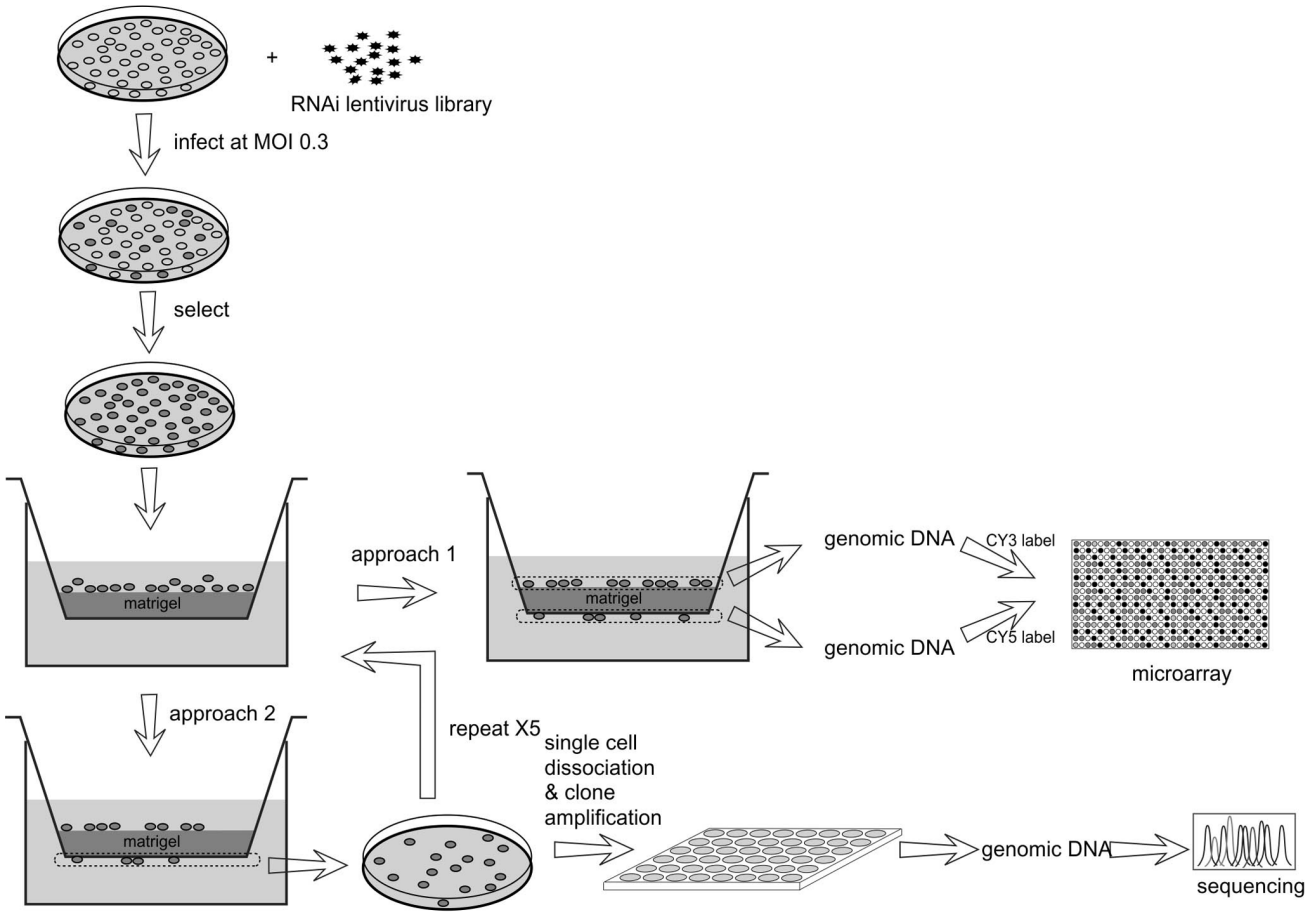
The multiplexed RNAi screening approaches. U87 cells were transduced with the lentivirus library. Following antibiotic selection, the cells were used for migration assay using a Matrigel invasion chamber. In approach 1, migrated and non-migrated cells were separately collected for genomic DNA extraction. The barcode region was amplified by PCR and labeled with CY3 or CY5, and used for microarray analysis to compare the shRNA abundance in either population. In approach 2, the migrated cells were collected and amplified, then subjected to another round of migration selection. The procedure was repeated 5 times before the final cells were used for single cell amplification in 96-well plates. After clonal expansion, genomic DNA was extracted and sequenced to determine the shRNA sequences in each clone.

The strategy of the multiplexed genome-wide RNAi screen is illustrated in **Figure 1**. To create the starting cell population, U87 cells were transduced with the Decode RNAi human annotated genome screening library (Open Biosystems). The library contains 3 pools of lentivirus containing a total of approximately 30,000 constructs, targeting 11,954 annotated human genes. For transduction, the virus to cell ratio was controlled to obtain approximately 100-fold coverage of each shRNA construct. To ensure that the majority of the cells have only one copy of the virus, a multiplicity of infection (MOI) of 0.3 was used so that only about 10% of the transduced cells had more than one copy of the virus. Following antibiotic selection to remove the non-transduced cells, we obtained a mixed cell population harboring 30,000 different shRNAs.

The transduced cells were loaded into a Matrigel invasion chamber, incubated for 12 hours, and then subjected to analysis by two different approaches. In approach 1, the migrated and non-migrated cells were separately collected to extract genomic DNA. The barcode region in the shRNA constructs was PCR amplified from the genomic DNA and labeled with either Cy3 or Cy5 dyes. They were then hybridized to a microarray with probes targeting the barcode sequences of the Decode library as described in the Methods. By comparing the Cy5/Cy3 signals at each spot, the abundance of individual shRNA in the migrated versus non-migrated cell population can be determined. Experiments were carried out in duplicate; the signals from all probes targeting the same construct in the two independent microarrays were averaged for assessing the effect of the shRNA. In approach 2, the migrated cells were collected, amplified, and then loaded for migration selection again. The procedure was repeated a total of 5 times until the migrated cells were dissociated into single cells for clonal expansion. In approach 2 the experiment was repeated once and from each experiment, we established 150 clones. Genomic DNA was then purified from each clone and the corresponding shRNA sequence was determined by sequencing.

The Cy5/Cy3 values for all the probed shRNA constructs are sort ordered and ranked in **Table S1**. Since the Cy5 and Cy3 signals at each spot should be proportional to the abundance of corresponding shRNA in the migrated and the non-migrated cell populations, this result provides an overall assessment for almost all the shRNAs on their effects on GBM cell migration. The Cy5/Cy3 ratio values were ranked from high to low and the ranking percentile was used for assessing the inhibitory effect of the shRNA on cell migration. This percentile translates to the percentage of shRNAs that have lower Cy5/Cy3 values than it is, so that a higher percentile represents a higher Cy5/Cy3 value. Hence, the targeting gene is more likely to inhibit GBM cell migration. In approach 2, a total of 300 clones were established and subjected to direct sequencing to determine the corresponding shRNA. Interestingly, only 29 difference constructs were identified, among which, 25 sequences appeared at least once in both experiments. The sequences and corresponding genes are shown in **Table 1**. This result suggest that the selection pressure was successfully applied, leading to effective enrichment of the adapted cells. However, it needs to be noted that in this approach, the selection pressure is not specific to the cell's migratory capability. shRNAs promoting cell proliferation may also be enriched as they give the cells an advantage during the *in vitro* amplification step. Indeed, not all of the 25 genes have high percentile in the results from approach 1 (**Table 1**). Since approach 2 also generated pure clones harboring the 25 shRNAs, we next used these clones for secondary screening to validate the effects of these primary hits on GBM cell migration.

**Table 1:** Genes identified in the RNAi screening.

Gene	Target sequence	Colony frequency	Inhibition ranking
FIGNL1	CCAGGAAACAGATAGTAAT	68 (22.7%)	44.6±8.8%
SENP8	CTGGCTCAATGACCATATT	39 (13.0%)	55.7±10.1%
LCTL	GAAACTTGCTCTATCAACA	33 (11.0%)	51.8±13.4%
VAV1	GGCAGAAATACATCTACTA	32 (10.7%)	N/A
HCFC1	CAACCACCATCGGAAATAA	20 (6.7%)	86.6±7.5%
GOLGA6L5	AGCTAAACATCACCATCAT	16 (5.3%)	N/A
B3GAT2	AAATAACTGCACTAAGGT	12 (4.0%)	87.9±7.3%
FLNA	CCTACTTTGAGATCTTTA	12 (4.0%)	N/A
KHSRP	CGAGAAGATTGCTCATATA	11 (3.7%)	95.5±1.0%
DLK1	CACATGCTGCGGAAGAAGA	8 (2.7%)	77.5±9.3%
PROKR1	CCTGGTCCGCTACAAGAAA	6 (2.0%)	94.3±4.1%
TERF1	GTAATGATGTTGAAATGGAA	6 (2.0%)	N/A
LRRIQ3	CTCACTTTAACTTACCAAA	5 (1.7%)	85.3±12.8%
TWF1	CAACTTGTGATTGGATCAT	4 (1.3%)	82.9±8.0%
NOB1	CTCCTGTGCATTTAATTAA	3 (1.0%)	90.3±8.8%
ERCC2	CTCACCGACTGCTTCCTGA	3 (1.0%)	N/A
RIPK1	ACCAACAGATGAATCTATA	2 (0.7%)	15.8±15.9%
HEPHL1	CCCAACAGGATAGGCAGTA	2 (0.7%)	41.1±14.7%
SMAD1	CTATTTCATCTGTATCTT	2 (0.7%)	85.3±11.1%
XPO4	CAGCGATTCTTAAGAGTGA	2 (0.7%)	52.9±17.3%
BUB1	CAGGAAAGGTCCGAGGTTA	2 (0.7%)	27.3±7.9%
AMMECR1	CTCCTTCCTTCCACATTTA	2 (0.7%)	80.9±6.5%
VPS18	CATTGTACGTGCTAAATGA	2 (0.7%)	33.6±11.4%
DUSP12	GTCGAAGTGTGGCCATAAT	2 (0.7%)	88.2±5.7%
CCNC	CTCCTTTCATGATAGCTTT	2 (0.7%)	68.5±10.0%

### Validation of the screening results *in vitro*

**Figure 2:**
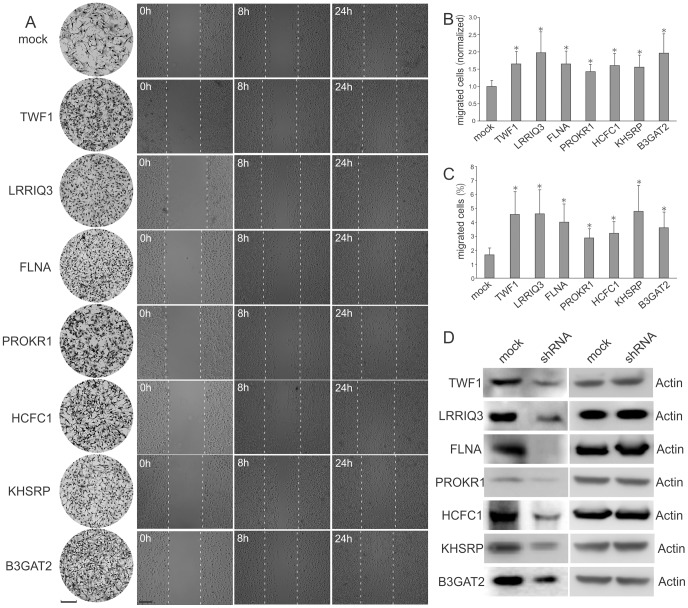
Identification of potent migration inhibiting genes. Seven genes from the screening hits were confirmed to affect U87 cell migration. (A) Column 1, a representative area showing the migrated cells attached to the lower membrane surface of the Matrigel invasion chamber. Cells were either mock transduced or transduced with shRNAs targeting the indicated genes. Columns 2 to 4, images showing cell migration in a wound healing experiment at indicated time points. Scale bar, 100 µm. (B) Quantification of the cell migration in the Matrigel invasion chamber experiments. n = 3. *, P<0.05. (C) Quantification of the cell migration in the wound healing experiments. n = 3. *, P<0.05. (D) Confirmation of the protein knockdown by western blotting.

Two independent cell migration assays were used to measure the migratory capability of the cell lines harboring the shRNAs we identified through RNAi screening. In the first assay, we used a Matrigel invasion chamber. After 8 hours of incubation, cells migrating to the lower surface of the membrane were stained for microscopic examination and compared to mock transduced cells produced by lentivirus harboring a scrambled shRNA sequence to determine the shRNA effect. Since U87 cells have strong migratory capability, usually thousands of cells were observed on the lower surface of the membrane. To accurately and reliably count the migrated cells, we developed an automated microscopic image processing program (**Method S1** and **Figure S1**). This tool enabled us to automatically quantify and statistically evaluate the results (**Figure 2A and B**). In the second measurement, we used a wound healing assay. A gap of approximately 250 µm was made by scratching with a pipette tip and the number of cells migrating across the border was monitored by time-lapse imaging. After 8 hours, cells exhibited different levels of migration until the gap was filled after 24 hours (**Figure 2A**). Overall, of the 25 cell lines we tested, 7 of them were observed to have significantly improved migratory capability in both assays (**Figure 2A, B and C**), suggesting an inhibitory role of the corresponding genes on cell motility. We mentioned above that some of the 25 primary hits did not rank high by approach 1; however, except for FLNA which has no corresponding probe on the array, the ranking percentiles of all the other 6 shRNAs confirmed by the two migratory assays are higher than 80% (**Table 1**), further supporting an inhibitory role specifically on cell migration. To further confirm this result, we also carried out experiments to directly exclude the effect of the shRNAs on cell proliferation. The proliferation of cell lines harboring the 7 shRNAs were monitored daily for 6 days and compared with mock-transduced cells. None of the gene knockdowns caused a significant cell proliferation change in U87 cells using either MTS assay or viable cell count measurement (**Figure S2**). In order to confirm the knockdown effects of the shRNAs on protein level, western blotting was carried out comparing the mock-transduced cells and the shRNA transduced cells. The results confirmed that the 7 shRNAs induced downregulation of the corresponding protein (**Figure 2D**). In summary, our screening identified 7 genes whose corresponding proteins may participate in the inhibition of GBM cell migration *in vitro*.

### Validation of the screening results *in vivo*

**Figure 3:**
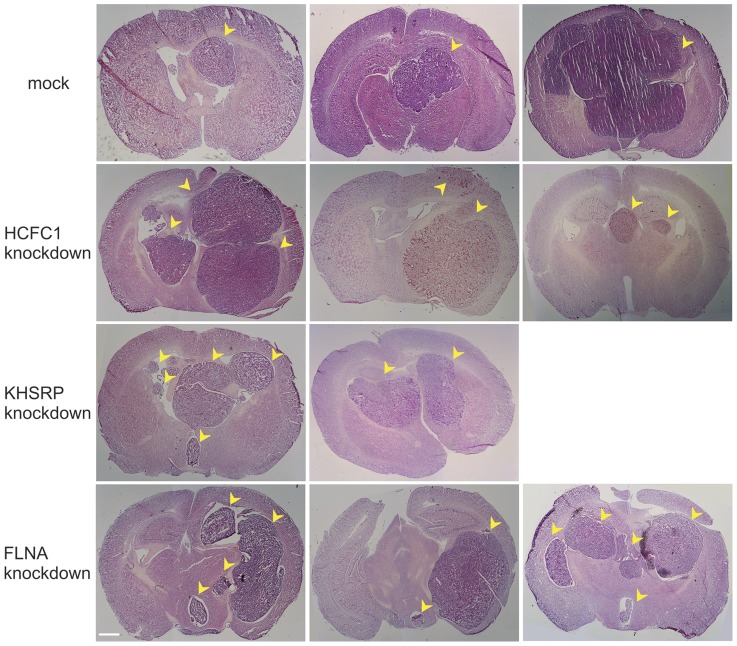
Multifocal brain tumor resulting from the gene knockdown. U87 cells were either mock transduced or transduced with shRNA virus before they were injected into the mouse brain. A total of 10 animals were used for each group. Mock transduced cells caused unifocal tumors varying in sizes (three examples were shown with small to big sizes). Knockdown of three genes resulted in multifocal tumors in some of the animals (3 animals for HCFC1 knockdown, 2 animals for KHSRP knockdown, and 3 animals for FLNA knockdown). Tumors are indicated by yellow arrow heads. Scale bar, 1 mm.

We further tested whether the 7 genes function *in vivo* to regulate GBM cell migration. U87 cells harboring the shRNAs were amplified for brain injection into immunodeficient mice. A total of 10 mice were injected for each cell line. All injections led to aggressive tumor growth in the animal brain and the animals died after approximately 1 month. No significant difference in the survival length was observed among all the cell lines tested (data not shown). After animal death, the brains were dissected for pathological examination. Standard H/E staining revealed tumor growth at the site where cells were injected, with a clear margin that differentiated them from the normal brain tissue (**Figure 3**). For mock transduced cells, although the resulting tumors varied in size significantly, they were all unifocal even that some tumors have invaded into the other hemisphere. Different pathology was observed for 3 of the 7 cell lines tested: those with shRNAs targeting genes HCFC1, KHSRP and FLNA; while the tumors for the other 4 cell lines are indistinguishable from the control tumors. For these three cell lines with shRNAs targeting HCFC1, KHSRP and FLNA, multifocal tumors were detected in some of the animals (**Figure 3**). The frequency of multifocal tumor was not high, occurring in 3 out of 10, 2 out of 10, and 3 out of 10 animals for HCFC1, KHSRP and FLNA cell lines, respectively. Multiple tumors were observed clearly separated from each other. The fact that some tumors were observed in the left hemisphere suggests that this separation is highly unlikely to be caused by technical reasons related to the injection procedure, rather it is a result of cell migration and amplification from the primary tumor. The fact that separation is not observed in any of the animals injected with mock transduced cells indicates that it is a result of gene downregulation, suggesting a role for genes HCFC1, KHSRP and FLNA in GBM cell migration *in vivo*.

### Validation of the gene effects with other GBM cells and secondary shRNAs

**Figure 4:**
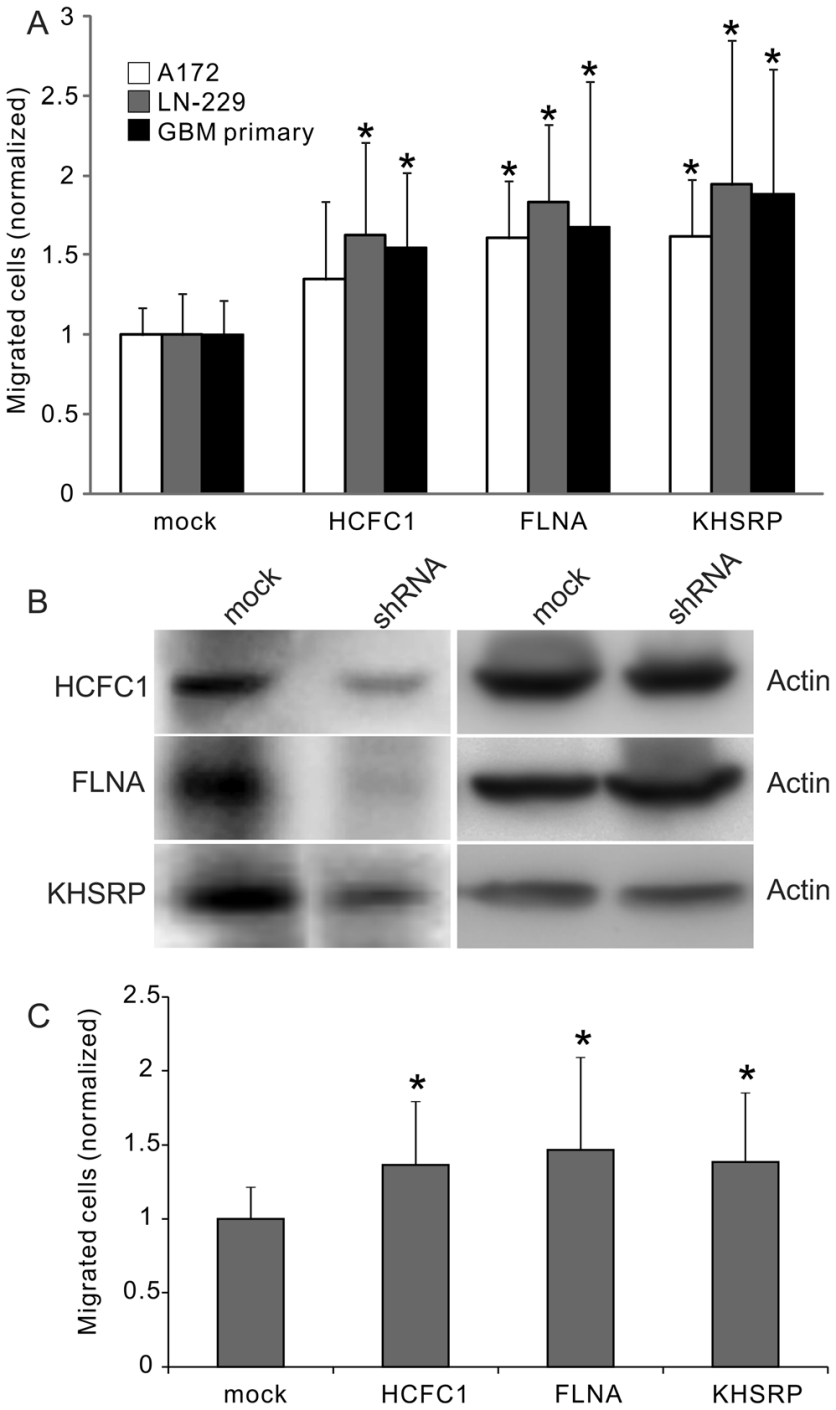
Validation of the gene effects with other GBM cells and secondary shRNAs. (A) The effect of the shRNAs on GBM cell lines A172, LN-229 and primary GBM tumor cells. Experiments were carried out using Matrigel invasion chamber. *, P<0.05, n = 3. (B) Protein expression change after the treatment of a secondary shRNA sequence targeting genes HCFC1, FLNA and KHSRP. (C) The effect of the secondary shRNAs on U87 cell migration. Experiments were carried out using Matrigel invasion chamber. *, P<0.05, n = 3.

The above screening and validation experiments were all carried out on U87 GBM cell line. In order to test whether the effects of HCFC1, KHSRP, and FLNA are general to GBM cells, two different GBM cell lines, A172 and LN-229, were used in the Matrigel invasion chamber experiment to further test the gene functions. In addition, primary GBM cells were cultured from patient surgical specimen and used for the cell migration assay. The primary cells were maintained in the neurosphere form (**Figure S3**). Before migration assay, they were dissociated into single cells to load into the Matrigel invasion chamber. After incubation, cells migrating to the lower surface of the membrane were stained and counted using the automated image processing program (**Figure S1**). Knockdown of the three genes led to significant increase of cell migration in all conditions except for HCFC1 in the A172 cell line, in which a higher average was observed but was not statistically significant (**Figure 4A**). These results support that HCFC1, FLNA, and KHSRP are inhibitory genes for the migration of GBM cells.

To verify the gene targets and avoid off-target effect, a secondary shRNA lentiviral construct was built and tested for genes HCFC1, FLNA and KHSRP. The sequences of the shRNAs were shown in **Table S2**. Western blot showed that these shRNAs were effective on down-regulating the corresponding proteins (**Figure 4B**), and Boyden Chamber migration experiment confirmed that the cells gained high motility after the down-regulation of the proteins (**Figure 4C**). This result verified that the effect of the shRNAs were through the targeted proteins.

### Association of the gene expression with clinical outcome

Tumor cell invasiveness directly contributes to the poor prognosis of GBM. In order to test whether the genes identified in this study are possibly involved in the tumor progression in patients, we sought to identify whether there is any association of the genes with the clinical outcome of GBM patients. For this study we used the most recent TCGA (The Cancer Genome Atlas) database, which contains data from 548 GBM patients. Interestingly, high expression levels of HCFC1 and KHSRP were observed for patients who survived long after surgery. Specifically, 70% of the patients who survived more than 3 years express higher than median level of HCFC1 as detected by the two probes targeting the gene. When the patients surviving more than 5 years were analyzed, even higher percentages were observed, with 91.1% and 83.3% of the patients above the median level as detected by the two probes, respectively. For KHSRP, approximately 70% of patients survived more than 3 or 5 years, as detected by 2 of the 3 probes targeting the gene (**Table 2** and **Figure S4**). Statistical analysis showed that the phenomenon is significant, supporting a possible role for HCFC1 and KHSRP in disease progression and suggesting that they may be used as novel prognostic markers for GBM patients.

**Table 2:** Correlation of patient survival length with HCFC1 and KHSRP expression.

	HCFC1	HCFC1	KHSRP	KHSRP	KHSRP
	probe 1	probe 2	probe 1	probe 2	probe 3
Total	50.0%	50.0%	50.0%	50.0%	50.0%
(548 patients)					
					
Survival >3 yrs	70.0%*	70.0%*	70.0%*	70.0%*	50.0%
(30 patients)	(p = 0.004)	(p = 0.002)	(p = 0.003)	(p = 0.013)	
					
Survival >5 yrs	91. 7%*	83.3%*	66.7%*	75.0%*	58.3%
(12 patients)	(p = 0.001)	(p = 0.007)	(p = 0.047)	(p = 0.027)	

Data are presented as the percentage of patients with expression above median level.

**Figure 5:**
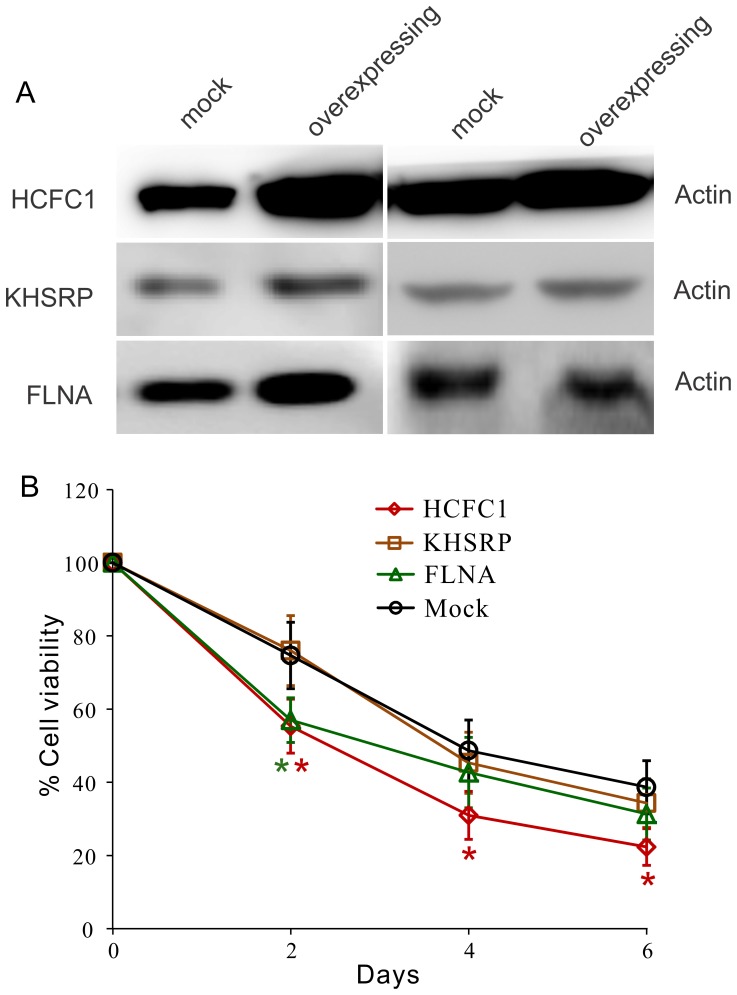
Effect of the gene overexpression on cytotoxicity response. (A) Cells were lentivirus transduced to overexpress the proteins of interest. (B) Cell viability after the treatment of 20 µM TMZ over 6 days. *, p<0.05, n = 3.

There are evidences suggesting that decreasing the migratory capabilities of tumor cells may sensitize them to cytotoxic reagents [9], [10]. Considering that most of the long survival patients received chemotherapy (87% of the patients survived longer than 3 years and 92% of the patients survived longer than 5 years), we sought to test if the high-expression of the genes can affect the chemotherapy efficiency. Cytotoxicity was measured every 48 hours over 6 days for the overexpressing U87 cells treated with 20 µM of temozolomide (TMZ). The result (**Figure 5**) showed that one of the cell line which overexpresses HCFC1 had enhanced cytotoxicity response at all the time points tested, while the other cell line overexpressing FLNA was observed to be sensitized to TMZ after 48 hours only. This result raises the possibility that the long survival may be not only caused by the decreasing of tumor cell migration, but also the enhancement of the chemotherapy efficiency, although more evidence is needed to draw the final conclusion.

## Discussion

Genome-wide RNAi screening has been increasingly used to study diverse biological processes.[23] Particularly, pooled shRNA screening is widely used because it has the advantages of low cost, high speed, and high coverage [24], [25]. This approach allowed us to carry out an unbiased study to systematically characterize all the human annotated genes for their affects on human GBM cell migration. Although a limited number of clones were sequenced, only 29 constructs were identified after sequencing 300 clones, highly suggesting that these shRNAs affect GBM cell migration. Also, the use of two different screening approaches and the consistency between the results further validate the effectiveness of our screening. Among the 7 screening hits that were confirmed by cell migration assays, 4 proteins were previously shown to interact with cytoskeleton or possibly be associated with cell migration. For example, FLNA (filamin) is known to crosslink actin filaments into orthogonal networks and participate in the anchoring of membrane proteins for the actin cytoskeleton [26], [27]. Interestingly, it was recently shown that FLNA can suppress breast cancer cell migration and invasion by regulating focal adhesion disassembly [28]. This is highly consistent with our finding of FLNA's inhibitory role in GBM cell migration. Another actin interacting protein identified is TWF1 (twinfilin-1), which binds to actin monomer and prevents assembly of the monomer into filaments [29], [30]. Twinfilin-1 may serve as a link between rapid actin filament depolymerization and assembly in cells, therefore regulating GBM cell migration. However, it is unclear how the inhibitory function is exerted. In fact, a recent RNAi screen has identified twinfilin to promote lymphoma progression, suggesting a role promoting cell motility [31]. Similarly, PROKR1 was previously shown to be involved in cell motility, but it stimulates lung cancer cell migration and promote metastasis [32], [33]. Further study is required to determine why the opposite function on cell migration was observed in this study. Different regulatory networks may be involved in various tissue types. The last gene is B3GAT2, which is involved in the synthesis of the human natural killer-1 (HNK-1) carbohydrate epitope, a sulfated trisaccharide involved in cellular migration and adhesion, particularly in the nervous system [34]. Our data confirms its role in cell motility in a tumor originating from brain.

Three genes were shown to be able to regulate GBM cell migration *in vivo* in an animal tumor model. The down-regulation of these genes significantly enhanced the migratory capability of GBM cells but no cell morphology or cytoskeleton structure change was detected (**Figure S5A, B**). Surprisingly, the cell-matrix interactions are changed divergently by the down-regulation of these genes – while the knocking-down of FLNA reduced the cell-matrix adhesion, the effects of the knocking-down of HCFC1 and KHSRP (**Figure S5C**) were enhancing. On the other hand, no effect on cell-cell adhesion was observed for the three genes (**Figure S5D**). These results suggest that although the cell motility effect of these genes are likely though regulating cell-matrix interaction, their mechanisms are different which remain to be further investigated. Among the three genes, FLNA is known to interact with actin as aforementioned. The other two genes, KHSRP and HCFC1, have not previously been reported to directly regulate cell motility. KHSRP encodes for a KH-type splicing regulatory protein, which is a multifunctional RNA-binding protein involved in mRNA decay and alternative pre-mRNA splicing. It promotes the rapid decay of AU-rich element (ARE)-containing mRNAs. Genes regulated by KHSRP were previously thought to be involved in cell proliferation, stress response, and cancer [35], [36], [37]. However, in our experiment, KHSRP did not affect U87 proliferation; thus, the enrichment of this gene in our screen is likely caused by other roles of the gene in GBM cells. The last gene, HCFC1, is also a well characterized gene encoding for host cell factor C1. It is well known to control the cell cycle and transcriptional regulation during herpes simplex virus infection [38]. There are indirect evidences suggesting that the protein may be involved in cell migration. First, structure analysis showed that the protein contains a fibronectin-like motif, implicating a role related to cell-matrix interaction. Second, HCFC1 is known to interact with CREB3, a protein previously shown to be involved in leukocyte migration [39], [40]._ENREF_37 This study further shows that the protein may have a role in cell migration regulation in processes other than virus infection.

Molecules affecting GBM cell migration has attracted much research interest, for its potential to be used as better diagnostic/prognostic markers, or design more effective targeted therapy. It has been shown that gene expression signatures in high-migratory glioma cells are directly correlated with short patient survival [4]. More recently, miRNA expression has been systematically characterized in migrating GBM cells, and miRNAs promoting cell migration has been discovered to be enriched in poor grade glioma [41], [42]. In our study, we find two genes (KHSRP and HCFC1) that are associated with the clinical outcome of long-surviving GBM patients. However, although most long-surviving patients have expression levels above the median values, high expression of the two genes do not necessarily lead to long survival length. This may be explained by the fact that the tumor progression state varied when the patients underwent surgical treatment, so that many patients may already have had extensive tumor invasion, even though they express high levels of inhibitory genes. The same reason may explain the fact that no significant correlation was observed on low expression of the two genes with short patient survival -because the survival time is counted as the days after tumor surgical removal other than the days after tumor initiation, the short-survival patients may actually be a mixture of patients carried tumors for various length. Nevertheless, expression levels of the two genes can be used clinically as supplemental indicators for patient survival prediction but not independent prognosis markers. The therapeutic application of the genes identified in this work needs to be further explored. In the past, research was focused on the identification of migration promoting genes so that potential treatment could be designed using inhibitors of the corresponding protein targets [43]. In order to translate the migration inhibitory mechanism to therapeutic strategy, further illustration of the complete pathways involved is required.

## Supporting Information

Figure S1 **Automated cell counting program.** (A) Raw image. (B) Image after processing, cells are labeled with different colors for clarity. (C) Magnified image of the box area in B, the accuracy of cell detection is over 95%. Particles on the membrane (an example pointed by red arrow) are excluded.
(TIF)

Figure S2 **The effect of gene knockdown on U87 cell proliferation.** Cells were infected with shRNA lentivirus targeting the indicated genes (or mock transduced) before experiments. Cell proliferation was monitored every 24 hours using two methods, MTS assay or viable cell count, for 6 days. Results were shown as the absorbance at 490 nm (A490) in MTS assay (left), or the number of viable cells counted (right). Experiments were repeated 6 times and results were shown as average with standard deviations.
(TIF)

Figure S3 **Primary culture of GBM cells.** (A) Fresh tumor samples were obtained within 2 hours of surgery. (B) Neurospheres form within 7 days in suspension culture in serum free medium containing bFGF. (C) After removing the attached cells as well as non-proliferating single cells, pure neurospheres were obtained.
(TIF)

Figure S4 **Association of HCFC1, KHSRP, and FLNA expression with patient survival length.** Data was collected from The Cancer Genome Atlas (TCGA) and analyzed for each probe corresponding to the genes of interest separately. A horizontal line was drawn at median expression level, a vertical line was drawn at 5 years survival length. For both probes of HCFC1, and probes 1 and 2 for KHSRP, significantly more patients surviving more than 5 years were observed with high expression level, as indicated by the red regions compared to the green regions. No significant differences were observed for other probes.
(TIF)

Figure S5 **The Effect of HCFC1, KHSRP, and FLNA knocking-down on cell morphology, cell-matrix adhesion and cell-cell adhesion.** (A) Phase contrast imaging shows no detectable cell morphology change after the down-regulation of HCFC1, KHSRP or FLNA. GFP expression shows that the shRNA treated U87 cells were successfully transduced. (B) F-actin structure of the U87 cells treated with shRNAs. Arrow pointed are focal adhesion structures. (C) Cell-matrix adhesion after the knocking-down of the three genes. *, p<0.05, n = 4. (D) Cell-cell adhesion after the knocking-down of the three genes.
(TIF)

Table S1 **Screening approach 1 result.** The Cy5/Cy3 ratio values from all the probes were ranked from high to low and the ranking percentile was used for assessing the inhibitory effect of the shRNA on cell migration. This percentile translates to the percentage of shRNAs that have lower Cy5/Cy3 values than it is, so that a higher percentile represents a higher Cy5/Cy3 value. The targeting genes for probes with high ranking are more likely to inhibit GBM cell migration
(XLSX)

Table S2 **Target sequences of the secondary shRNAs**
(DOCX)

Method S1 **Image processing pipeline**
(DOCX)
