# Supplementary figures and images for: Genome-Wide RNAi Screening Identifies Genes Inhibiting the Migration of Glioblastoma Cells
Source: PLoS One. 2013 Apr 12;8(4):e61915. doi: 10.1371/journal.pone.0061915 (PMC3625150; doi:10.1371/journal.pone.0061915)

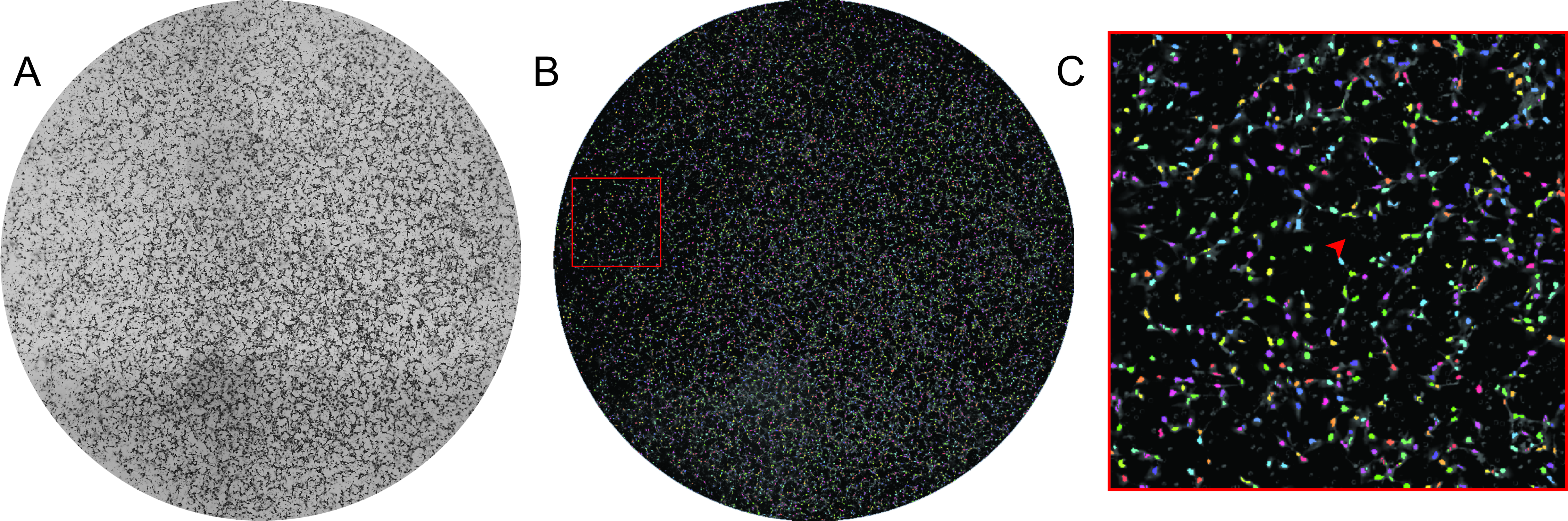

Supplement: Figure S1 — Automated cell counting program. (A) Raw image. (B) Image after processing, cells are labeled with different colors for clarity. (C) Magnified image of the box area in B, the accuracy of cell detection is over 95%. Particles on the membrane (an example pointed by red arrow) are excluded. (TIF) [file pone.0061915.s001.tif]

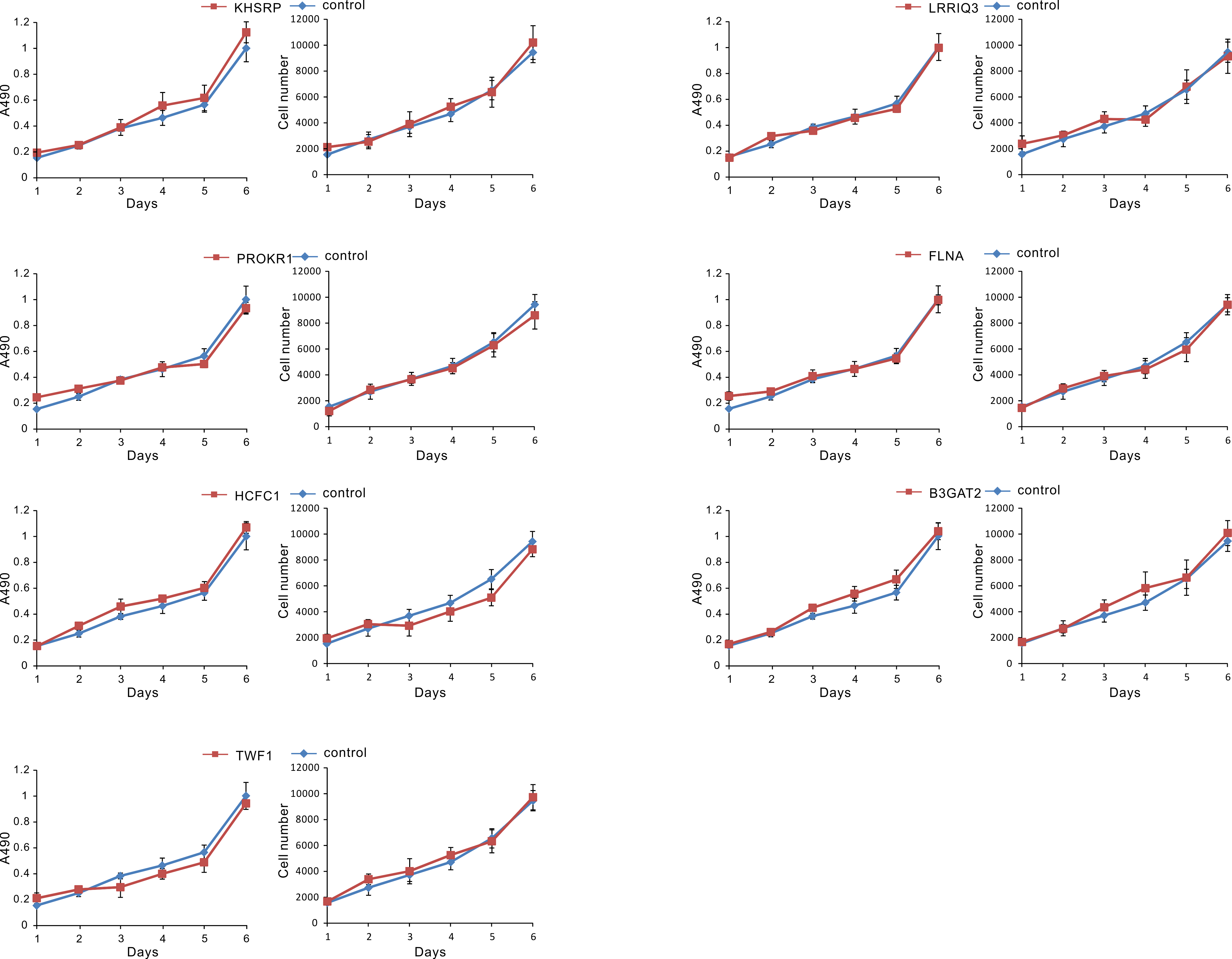

Supplement: Figure S2 — The effect of gene knockdown on U87 cell proliferation. Cells were infected with shRNA lentivirus targeting the indicated genes (or mock transduced) before experiments. Cell proliferation was monitored every 24 hours using two methods, MTS assay or viable cell count, for 6 days. Results were shown as the absorbance at 490 nm (A490) in MTS assay (left), or the number of viable cells counted (right). Experiments were repeated 6 times and results were shown as average with standard deviations. (TIF) [file pone.0061915.s002.tif]

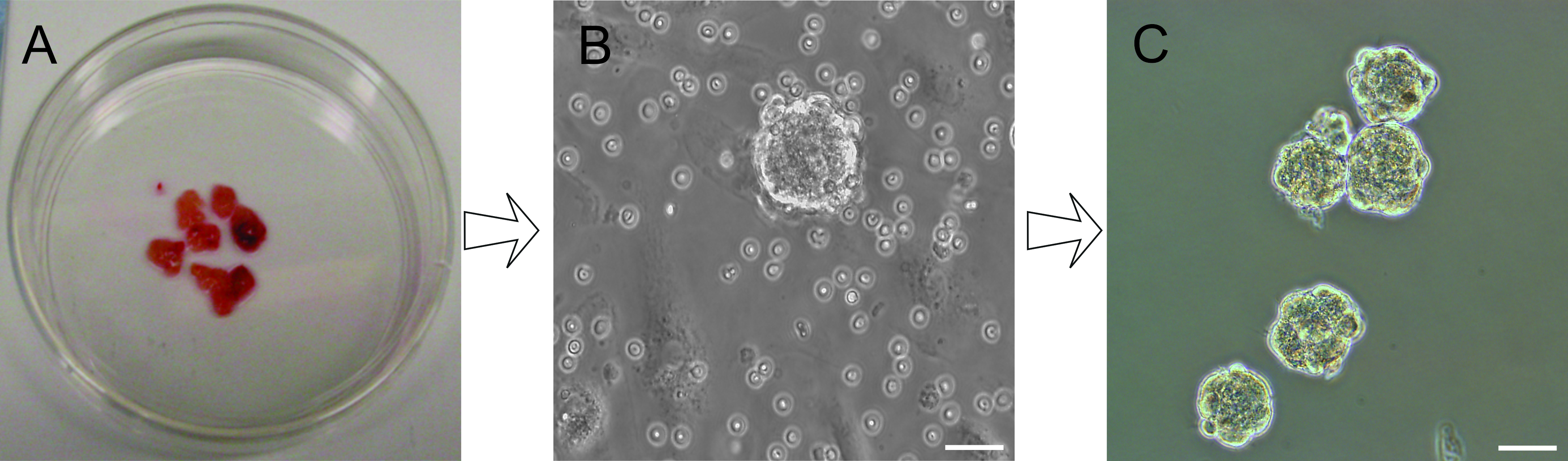

Supplement: Figure S3 — Primary culture of GBM cells. (A) Fresh tumor samples were obtained within 2 hours of surgery. (B) Neurospheres form within 7 days in suspension culture in serum free medium containing bFGF. (C) After removing the attached cells as well as non-proliferating single cells, pure neurospheres were obtained. (TIF) [file pone.0061915.s003.tif]

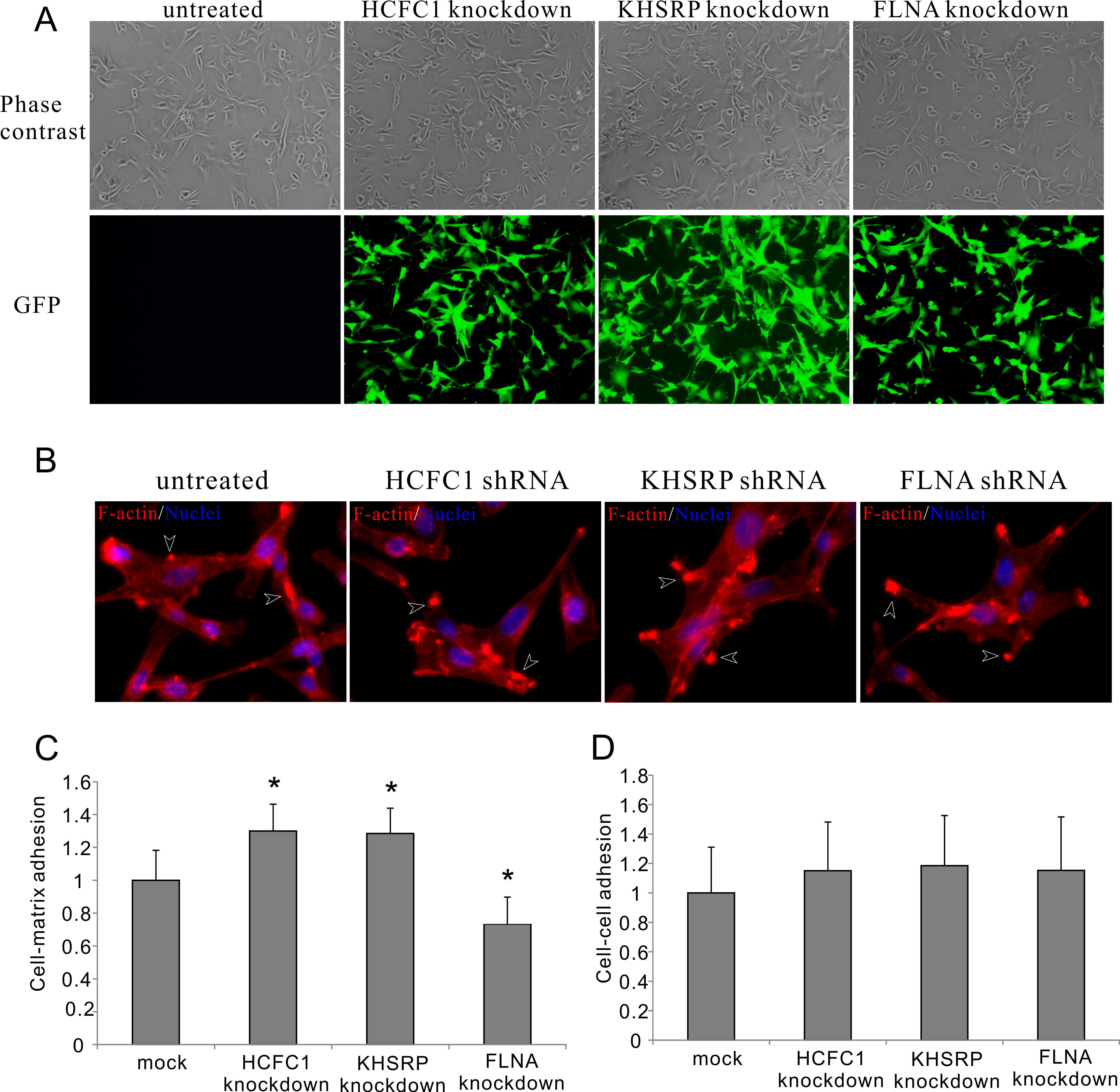

Supplement: Figure S5 — The Effect of HCFC1, KHSRP, and FLNA knocking-down on cell morphology, cell-matrix adhesion and cell-cell adhesion. (A) Phase contrast imaging shows no detectable cell morphology change after the down-regulation of HCFC1, KHSRP or FLNA. GFP expression shows that the shRNA treated U87 cells were successfully transduced. (B) F-actin structure of the U87 cells treated with shRNAs. Arrow pointed are focal adhesion structures. (C) Cell-matrix adhesion after the knocking-down of the three genes. *, p<0.05, n = 4. (D) Cell-cell adhesion after the knocking-down of the three genes. (TIF) [file pone.0061915.s005.tif]
