# Supplementary material for: Genome-Wide RNAi Screening Identifies Genes Inhibiting the Migration of Glioblastoma Cells
Source: PLoS One. 2013 Apr 12;8(4):e61915. doi: 10.1371/journal.pone.0061915 (PMC3625150; doi:10.1371/journal.pone.0061915)
Supplement: Table S2 — Target sequences of the secondary shRNAs (DOCX) [file pone.0061915.s007.docx]

**Table S2 Target sequences of the secondary shRNAs**

| **Gene** | **Target sequence** |
| --- | --- |
| HCFC1 | gtcctgtgactcagatcat |
| FLNA | gtccctgtgcatgatgtga |
| KHSRP | gtccagaaagccaagatga |
