# Supplementary material for: Genome-Wide RNAi Screening Identifies Genes Inhibiting the Migration of Glioblastoma Cells
Source: PLoS One. 2013 Apr 12;8(4):e61915. doi: 10.1371/journal.pone.0061915 (PMC3625150; doi:10.1371/journal.pone.0061915)
Supplement: Method S1 — Image processing pipeline (DOCX) [file pone.0061915.s008.docx]

**METHOD S1**

An automated pipeline was developed to detect and count the migrated cells on the membrane. The pipeline starts with Multi-Scale Variance Stabilizing Transform (MSVST) followed by an iterative adaptive thresholding on both the intensity and the size of objects. MSVST was originally proposed as a Poisson noise reduction strategy; we found it also effectively enhances signals in an inhomogeneous image. We integrated the VST and Undecimated Wavelet Transform (UWT) procedures by stabilizing the approximation coefficients $a_{j}$using VST and computing the wavelet coefficients from the stabilized $a_{j}$. The distribution of $w_{j}$is known as Gaussian, so we used classical hypothesis testing to detect the significant coefficients. The final estimates were then reconstructed with the detected coefficients. The detailed iterative reconstruction procedure is described in the following box.

With the MSVST enhanced map, we then used an adaptive thresholding procedure to segment cells from the inhomogeneous background. The strategy employed local information to identify cells in an iterative manner. First, a loose initial intensity threshold $T_{I}=k\bar{I}_{N}$ was applied to the enhanced image, $\bar{I}_{N}$is the average intensity of all objects in the neighborhood region with size N, k is a constant. For our images, $k=2$ and $\bar{I}_{N}$ = the average intensity of the whole image were used for the first iteration, and $k=0.9$is set for the rest iterations. Then, on the binarized image, we applied an area threshold$T_{A}$ to obtain regions larger than$T_{A}$ for further processing. For each region, an updated intensity threshold with $T_{I}=k\bar{I}_{N}$ was applied. If$T_{I}$ shrunk a region small enough or split the original region to smaller than the updated area threshold $T_{A}$, the shrunken region or split subregions were saved and considered final segmented spots. For subregions larger than the updated$T_{A}$, the process was repeated until the conditions were satisfied. An example of cell detection and counting using the above pipeline is shown in **Figure S1**.
